# Overcoming chemoresistance to b-raf inhibitor in melanoma via targeted inhibition of phosphoenolpyruvate carboxykinase1 using 3-mercaptopropionic acid

**DOI:** 10.1080/21655979.2022.2080385

**Published:** 2022-06-05

**Authors:** Ming Ren, Lu Wang, Zi-Xu Gao, Xin-Yi Deng, Kang-Jie Shen, Yan-Lin Li, Yi-Teng Ding, Chuan-Yuan Wei, Jian-Ying Gu

**Affiliations:** Department of Plastic Surgery, Zhongshan Hospital, Fudan University, Shanghai, China

**Keywords:** B-raf, resistance, melanoma, PCK1, 3-MPA, ROS

## Abstract

The resistance of melanoma to BRAF inhibitors remains a tough clinical challenge. In order to explore the underlying mechanism of drug resistance in melanoma, we established the resistant cell line to vemurafenib, and assessed the changes of drug-resistant cells on proliferation, apoptosis, oxidative stress and tumor stemness. Our results suggest that phosphoenolpyruvate carboxykinase1 (PCK1) is activated and inhibits the oxidative stress caused by vemurafenib in drug-resistant cells. Long term treatment of vemurafenib increases the expression of PCK1 which reduces the production of reactive oxygen species (ROS) by activating PI3K/Akt pathway. After the inhibition of PCK1 by 3-mercaptopropionic acid (3-MPA), the therapeutic sensitivity of vemurafenib is restored. In conclusion, this study disclosed that drug-resistant cells appeared to regulate their own proliferation, oxidative stress and tumor dryness by activating Akt/PCK1/ROS pathway, and shed new insights into acquiring drug resistance in melanoma.

## Graphical Abstract

**Figure:**
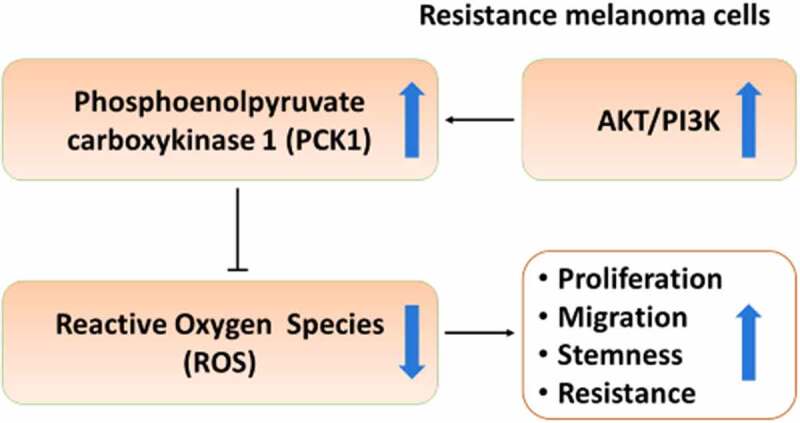

## Highlights

- PI3K/Akt pathway upregulated PCK1 in the resistant melanoma cells.
- PCK1 promoted proliferation and stemness, and reduced oxidative damage.
- 3-MPA sensitized vemurafenib by targeting PCK1, and suppressed the resistance via the Akt/PCK1/ROS axis.

## Introduction

The incidence of melanoma, a malignant tumor of melanocytes that arises in the skin, has been increasing in recent years[1]. BRAF mutation is a prominent feature of melanoma, which activates the downstream kinase MEK/ERK within the MAPK pathway, promoting melanoma progression[2]. Inhibitors designed to target the BRAF mutation, such as vemurafenib, dabrafenib and combined therapy of dabrafenib and trametinib, have achieved pioneering results[3]. However inevitable acquired resistance is the major factor contributing to the treatment failure, innovative strategies are still urgently needed to improve the treatment of melanoma therapy.

Metabolic reprogramming is closely related to melanoma occurrence, development, metastasis, drug resistance, and recurrence [4,5]. PCK1, a cytoplasmic form of PEPCK, is a rate limiting enzyme, which decarboxylate in the second step of gluconeogenesis after the carboxylation of pyruvate, and then phosphorylates oxaloacetic acid to form phosphoenolpyruvate (PEP) [6,7]. As a central molecule regulating glycolysis, the tricarboxylic acid cycle (TCA), and gluconeogenesis, PCK1 plays importantly biological effects [8]. Specifically, PCK1 is known to have a major role in generating abundantly NADPH to reduce the ROS through the Pentose Phosphate Pathway (PPP), which promotes the progression of colon and lung cancer [9]. The inhibition of PCK1 leads to metabolic imbalances, which hindering cell viability of transformed embryonal kidney and colon carcinoma tumor growth [10]. 3-MPA, a known inhibitor of gluconeogenesis, can inhibit PCK1 selectively, and shows a good performance in antitumor activity [11,12].However, the mechanism of PCK1 in melanoma remains unknown and regulation of metabolic reprogramming may be an Achilles’ heel of chemoresistance.

In this study, we aimed to investigate the potential role of PCK1 in regulating acquired drug resistance in melanoma. Additionally, the effect of 3-MPA on sensitizing vemurafenib was validated by assays *in vivo and in vitro.*

## Materials and methods

### Establishment of resistant melanoma cells to vemurafenib

A2058 cells were inoculated in a culture flask, and a high dose of vemurafenib (PLX4032, Cat#HY-L032) (MedChemExpress, USA) was used for treatment. When most of the cells had died, the dead cells were washed away and the medium was changed to a drug-free medium. Post reaching confluence, the cells were digested and re-inoculated into a new culture flask, and some cells were cryopreserved for RNA-seq. The cells were treated with high density vemurafenib until the cells reached 30% confluence. This process was repeated 3 times, and we obtained R0, R1, R2, and R3 cells, which were then used for RNA-seq analysis. Melanoma cells in the exponential growth phase were inoculated into culture bottles. After 48 h, the cells were transferred to a drug-free medium and expanded until the next mitotic phase. Then, the above steps were repeated using vemurafenib that was two times more concentrated. At the same time, cell death was observed every day, the fresh complete medium containing vemurafenib was replaced, and Cell Counting Kit 8 (CCK8) analysis was performed regularly until melanoma cells grew stably in the medium. This process lasted for six months, and transcriptome sequencing was performed once every two months; thus, 4 samples (R0, R1, R2, and R3) were obtained.

### Knock down and overexpression

According to the instructions, Lipofectamine 3000 (American life technologies) was used for cell transfection. After 72 hours, western blot was used to detect the transfection efficiency. siPCK1, pLVX-shRNA-eGFP-PGK-Puro and CMV-H_PCK1-eGFP-3flag-PGK-Puro lentiviral vectors were purchased from Genomeditech (Shanghai, China). In short, A2058 cell lines were planted into 6-well plates and transfected at 5 × 10^5^ cells per well (20 nM siRNA or shRNA). After 24 hours of transfection, the cells were replaced with fresh and complete culture medium, and the cells were collected for follow-up experiments (lentivirus group needed to be screened by 2 ug/ml puromycin). The target sequences were as follows: siPCK1 GCACATCCCAACTCTCGATTT.

### CCK8 cell proliferation assay

Cells were seeded in to a 96-well plate at a density of 4000 cell/well and cultured in a humidified cell culture incubator. After 24 hours, therapeutic drugs were added to the plate and continue to culture for 72 hours. 10 μL CCK8 reaction solution (Beyotime, China) was added to the cell culture at indicated time point and incubated for 3 h in cell culture incubator. The light absorption value (Optical Density value, OD) in each condition was measured at 450 nm wavelength on a Synergy H1 microplate reader (Winooski, Vermont, USA). The cell viability was calculated using the following formula: (OD of dosing well – OD of culture medium)/ (OD of control well – OD of culture medium) × 100%.

### Calculation of resistance index (RI)

The 50% inhibitory concentration (IC50) calculator (https://www.aatbio.com/tools/ic50-calculator) was used to prepare the survival rate and drug concentration curves, and the best fitting IC50 was calculated. The statistical figure of the IC50 value was drawn by GraphPad prism 6 software. The following formula was used: RI = IC50 of resistant cells/IC50 of parental cells.

### Cell migration and trans-well invasion assay

Ability of migration and invasion were examined by wound healing assay and trans-well assay respectively, and they were conducted as reported previously [13].

### Western-blotting analysis and antibodies

The total protein was obtained from all cells using RIPA buffer (Sigma, USA), and then the protein was quantified by BCA Kit (beyotime, China). Subsequently, 10 µg of protein was separated using 10% sodium dodecyl sulfate polyacrylamide gel, and then electrically transferred to polyvinylidene fluoride membrane, blocked with 5% skimmed milk for 2 hours, and incubated with primary antibody at 4°C for 8 hours, next, the membrane was incubated with secondary antibody at 37°C for 1 hour. Finally, the protein bands were observed by ECL system (Thermo, USA). All the antibodies used are listed in supplementary table 1.

### Cell apoptosis analysis

Flow cytometry was performed as reported previously [14] and the Annexin V Apoptosis Detection Kit APC was obtained from eBioscience. The specific gating strategies applies to supplementary material.

### ROS detection in cells and tissues

CellROX Regent orange solution was used to culture or cover the tissue and avoid light during the whole process. After incubation for 30 min at 37°C, we used a High Content Imaging System to observe the production of ROS in the cells. The dye solution was removed from the tissues and they were fixed. Then, photos were taken under a microscope, and the tissues were stored.

### Immunohistochemistry

The tumor tissues were dehydrated and fixed in a 10% formalin buffer solution for 24 h. The frozen sections were embedded in OCT, frozen, and continuously sectioned to 15 μm sections at 20°C. Immunohistochemistry was performed as previously described. The primary antibodies used are listed in Table S1.

### Animal models

Parental A2058 cells or drug-resistant melanoma A2058R cells (1 × 10^6^) in 200 μl of PBS were subcutaneously transplanted into SPF BALB/c nude mice (purchased from Zhongshan Hospital Laboratory). After 7–10 days of transplantation, the palpable tumor size (40–100 mm) was reached, and mice with a similar tumor size were randomly assigned to different four treatment groups. The parallel group design was adopted, and the mice were treated with 30 mg/kg vemurafenib, and/or 3-mercaptopicolinic acid (SKF-34288 hydrochloride, Cat#320,386-54-7) (MedChemExpress, USA) [15], and equal DMSO, once a day for 21 days. All animal protocols were approved by the animal protection and the use committee of Zhongshan Hospital, Fudan University.

### Calculation of the combination index

CompuSyn is available for download from http://www.combosyn.com. According to the Chou-Talalay mathematical model of drug interaction, the combination index (CI) of cells receiving combination therapy was calculated. Chou-Talalay CI theorem provides a quantitative definition of the additive effect (CI = 1), synergistic effect (CI < 1), and antagonistic effect (CI > 1) of drug combination [16].

### Statistical analysis

Each experiment was repeated three times. The results were shown by the mean ± standard deviation. SPSS software 19.0 was used for the statistical analyses, including Student’s t-test (two-tailed), Pearson’s correlation analysis, and the Log-Rank test. The significance threshold was set at 0.05 for each test.

## Results

PCK1 was a highly expressed protein in drug-resistant melanoma cells. In this study, we investigated the potential role of PCK1 in modulating the resistant phenotype of malignant melanoma. Our study revealed that overexpression of PCK1 promoted cell proliferation, invasion and tumor stemness, and reduced the oxidative damage induced by vemurafenib. After using 3-MPA, a specific inhibitor to PCK1, acquired drug resistance to vemurafenib could be relieved. The role of PCK1 and 3-MPA were validated in the mouse xenograft model.

### Establishment and phenotype of the drug resistance model

**Figure 1.**
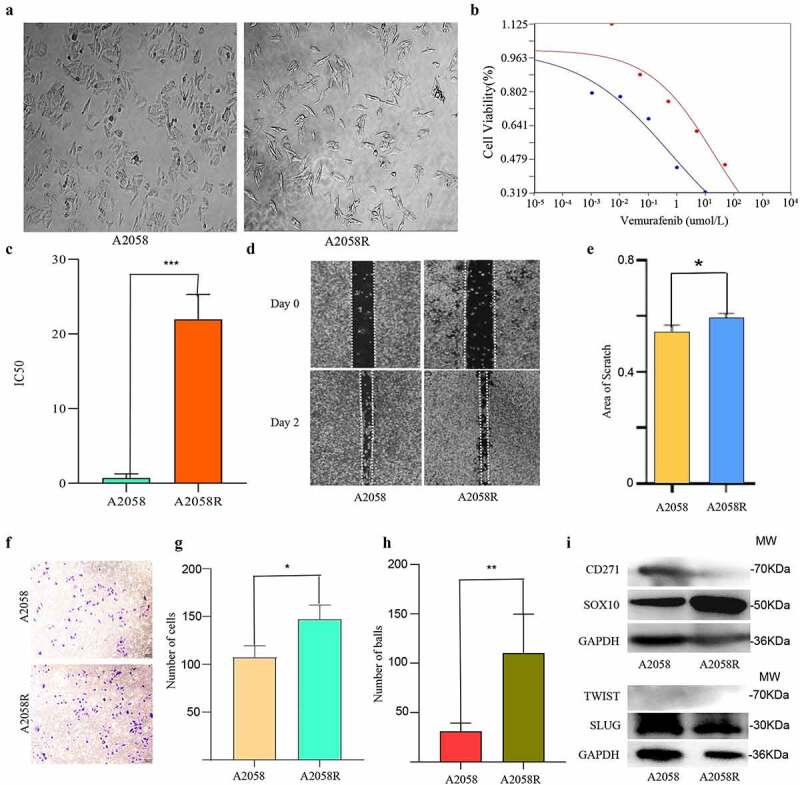
Establishment of a drug resistance model and the results of a phenotype test.

To investigate the molecular mechanisms underlying resistance of b-raf inhibitor, we exposed the melanoma cell line A2058 harboring the BRAF V600E mutation to high concentrations of vemurafenib. After 6 months, we obtained the resistant cell line A2058R. We found that the cells became slender under the action of vemurafenib at a high concentration (Figure 1a, 1b). Results of the CCK8 experiment confirmed that the IC_50_ of drug-resistant A2058R cells was higher than that of primary A2058 cells, (A2058 IC50 = 0.71, A2058R IC_50_ = 21.95, RI = 30.9) (Figure 1c, 1d). Scratch and trans-well assays confirmed that A2058R cells have greater invasion and migration abilities than A2058 cells (Figure 1e–1i). Meanwhile, WB assays showed that the levels of CD271, SOX10, TWIST, and SLUG were elevated in A2058R cells, indicating that they possess higher stemness and abilities of Epithelial-to-Mesenchymal Transition (EMT).

A. Wild type A2058 cells and A2058R cells resistant to vemurafenib. B. Cell viability curve shows that A2058R has higher viability. C. IC_50_ statistical plot shows A2058R possessed higher resistance (*** *p* < 0.001) D. Scratch assay E. Statistical figure of the scratch: A2058R has stronger proliferation ability (* *p* < 0.05). F. Transwell assay of A2058 cells. G. Results of the trans-well assay. H. Sphere forming experiments: A2058R has stronger ability of sphere forming (** *p* < 0.01). I. Western blot: A2058R cells shows a higher expression of SOX10, but there is no difference in TWIST and SLUG.

### PCK1 protein and AKT/PI3K signaling were key factors in the transcriptome sequencing results

**Figure 2.**
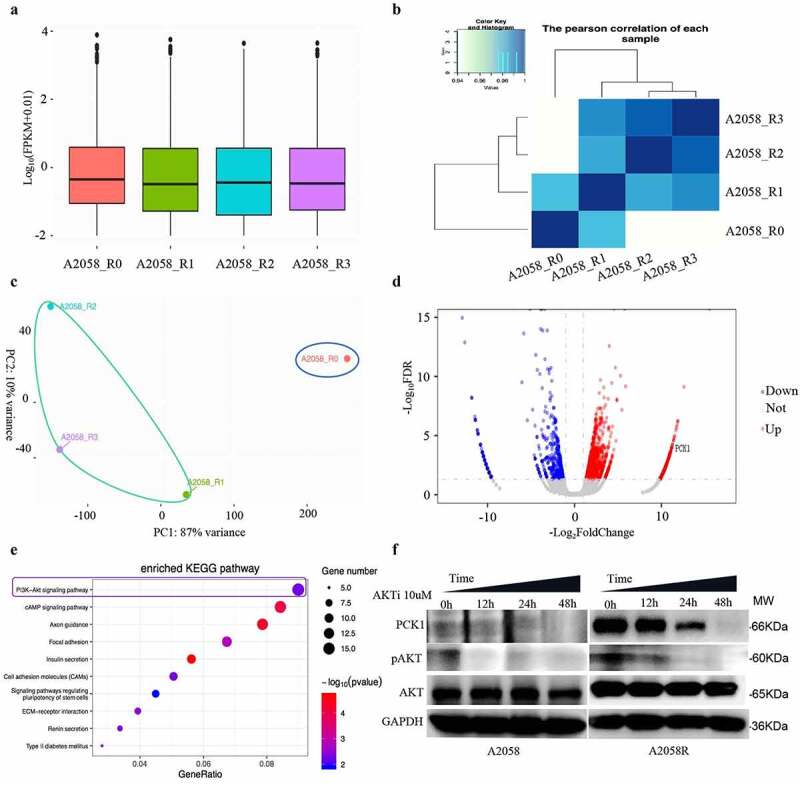
Identification and validation of the target molecules and signaling pathways using transcriptome sequencing.

After normalizing the transcriptome sequencing results (Figure 2a), we found that there were differences in the gene expression profiles among the four samples (Figure 2b), which were further corroborated by PCA results (Figure 2c). Differential expression analysis was performed, and we focused on *PCK1*, a gene whose expression level was high and constant in R1, R2, and R3, and we considered it as the key target gene for conferring of drug resistance (Figure 2d). In KEGG enrichment analysis (Figure 2e), we observed that the PI3K/AKT signaling pathway molecules were continuously highly expressed in the three drug-resistant samples. Combining with the related literatures ,,PCK1 and PI3K/Akt signaling pathways were involved in the process of acquired drug resistance induced by b-raf inhibitor in melanoma cells. Furthermore, western blot analysis confirmed the reasoning. In addition, when we inhibited the PI3K/Akt pathway, the expression of PCK1 decreased, suggesting that PCK1 is a crucial downstream molecule drug resistance (figure 2f).

A. Statistical plot: R0 represents wild-type sample; R1, R2, and R3 represent the samples collected during the construction of the resistant model. B. Pearson test: Pearson correlation suggests that the gene expression was variable for each sample. C. PCA analysis: the expression data is constitutively different between the wild-type and resistant cells. D. Volcano plot: the red presented the genes up-regulated and the blue were downregulated. The higher the position, the greater the difference. E. KEGG: the size of the circle represents the number of related genes, the color represents the statistical difference, the redder, the more statistical difference. F. Western blot: the expression level of PCK1 and p-AKT in A2058R cells is higher than that in A2058 cells, indicating that AKT was activated in A2058R cells. After the addition of AKT inhibitor, the p-AKT level gradually decreased, accompanied by the decrease in the PCK1 expression.

### PCK1 activity determined melanoma cell resistance to vemurafenib

**Figure 3.**
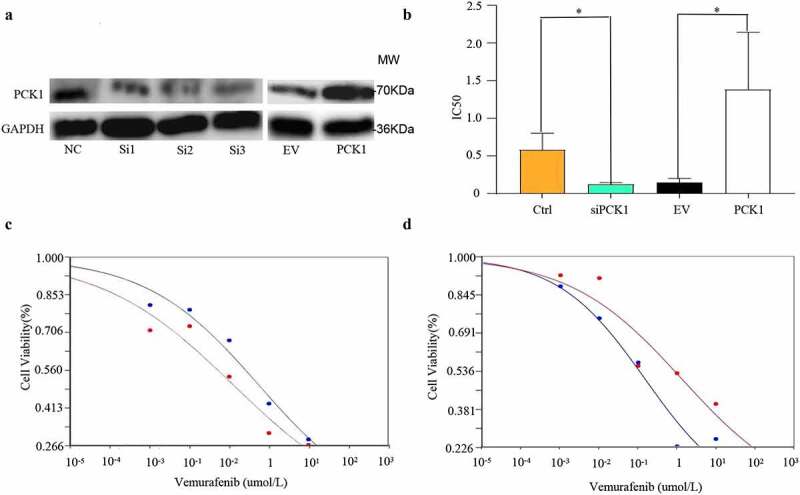
Effect of PCK1 on the chemoresistance in melanoma cells.

Next, we constructed low expression and overexpression PCK1 cell lines using siRNA and overexpression viruses, respectively. Western blot results indicated that the transfection was successful (Figure 3a). After PCK1 knockdown, cell viability significantly reduced, thus, the IC50 of siPCK1 and the resistance index (RI) both decreased (RI = 4.46), *P*< 0.005 (Figure 3b, 3c).

A. Western blot: the melanoma cell lines with low expression and overexpression of PCK1 were constructed successfully. B. IC50 figure: Knockdown of PCK1 shows low cell viability under vemurafenib, and overexpression shows high (* *p* < 0.05). C. Cell viability curve: the blue refers to the control, and the red refers to the knockdown (siPCK1). D. Cell viability curve: the blue represents the empty virus (EV), and the red represents the overexpression (PCK1).

However, the cell viability and resistance index (RI) were higher (RI = 9.27), *P*< 0.005, in the cell line overexpressing PCK1 (Figure 3b, 3d). Therefore, the results indicated that PCK1 was a crucial molecule that could enhance the tolerance of melanoma cells to vemurafenib.

### PCK1 promoted the proliferation, migration, and stemness of melanoma cells with V600E mutation

**Figure 4.**
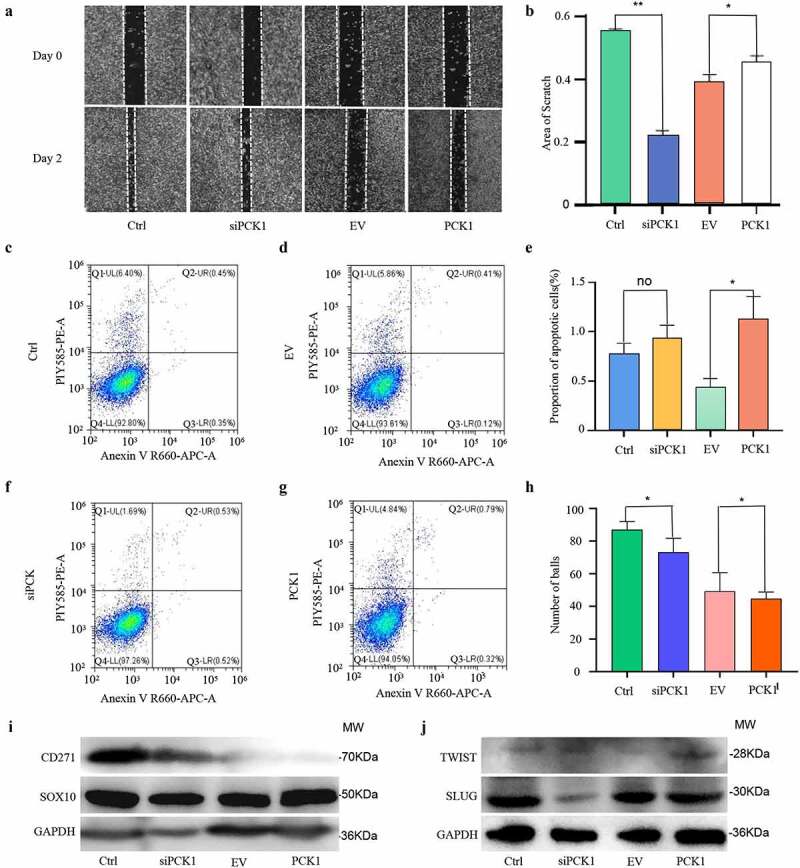
Functions of PCK1 in melanoma proliferation, migration, apoptosis, stemness, and EMT.

Based on the hypothesis that the development of drug resistance -might be caused by related genes that induce cancer stemness, EMT, and apoptosis inhibition, we first designed scratch and invasion experiments and found that the *PCK1* gene status was related to the proliferation and invasion of melanoma cells, while the activation of PCK1 could promote drug resistance in melanoma and increase the severity of the disease (Figure 4a, 4b). Next, flow cytometry was conducted, and the results showed that the knockdown and overexpression of PCK1 did not have a direct effect on apoptosis, although statistical differences were noted. This suggested that PCK1 might indirectly induce BRAFi resistance through PPP (Figure 4c–4g). In addition, the results of the tumor spheroidization test on a low adhesion plate showed that the spheroidization ability of A2058R cells was significantly higher than that of A2058 cells (Figure 4h), that of the SiPCK1 group was decreased, and that of the OE group was restored after the overexpression of PCK1. Combined with the western blot results, these results suggested that CD271 and SOX10, which have been recognized as indicators of melanoma stemness, were highly expressed in A2058R and OE cells (Figure 4i), indicating that PCK1 could modify cancer stemness in the development of drug resistance. Meanwhile, in this experiment, PCK1 could not alter the EMT of melanoma for achievement of drug resistance (Figure 4j). These lines of evidence illustrated that the activation of PCK1 potentiated melanoma proliferation, migration, and tumor stemness, all the phenotypes associated with drug resistance. The results revealed that PCK1 did not contribute to drug resistance directly by inhibiting apoptosis but, most likely, contributed indirectly to acquired drug resistance.

A. Scratch assay. B. Results of the scratch assay: the proliferation area of SiPCK1 cells is smaller than the control, and the proliferation area of PCK1 group is higher than that of EV. C. Flow cytometry of apoptosis: the Q2 + Q3 is the proportion of apoptotic cells. The percentage of apoptotic cells is 0.9% in the control group. D. Flow cytometry of apoptosis: the percentage of apoptotic cells is 0.53% in the EV group. E. Flow cytometry plot: there is no significant difference in the distribution of apoptotic cells. F. Flow cytometry plot: the percentage of apoptotic cells in the siPCK1 group was 1.05%. G. Flow cytometry plot: the percentage of overexpression of the PCK1 group was 1.11%. H. Results of the sphere formation assay: counting the number of cell spheres with a diameter larger than 50 µm under a field of view. I. Western blot: proteins related to temness. J. Western blot: proteins related to EMT.

### Synergistic effect of PCK1 inhibitor 3-mercaptopicolinic acid combined with vemurafenib on drug-resistant cells

**Figure 5.**
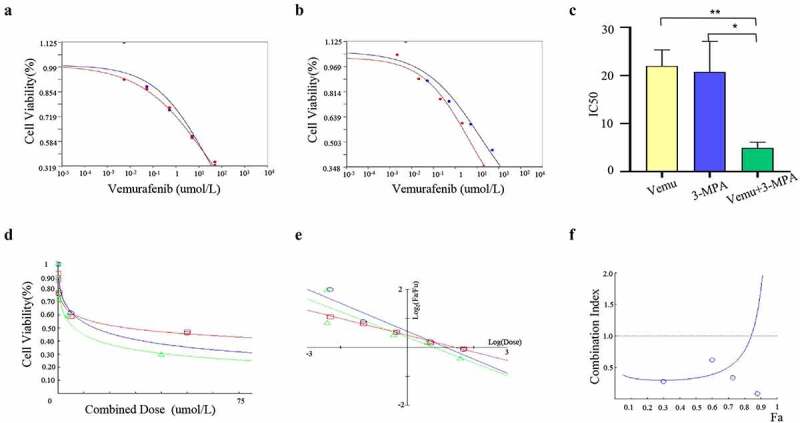
Synergism of PCK1 inhibitor 3-mercaptopicolinic acid in combination with vemurafenib in resistant cells.

The CCK8 assay was used to detect the activity of A2058R cells under different drug treatment regimens. The IC50 of A2058R cells treated with 3-mercaptopicolinic acid or vemurafenib alone did not change significantly (Figure 5a, 5c). However, the IC50 of 3-mercaptopicolinic acid combined with vemurafenib was significantly lower than that of vemurafenib alone, which indicated that 3-mercaptopicolinic acid increased the resistance inhibition of vemurafenib (Figure 5b, 5c). We used the Chou-Talalay method to compare the efficacy of each treatment group. IC50(3-MPA+vemu) = 6.37, IC50(vemu) = 14.47, IC50(3-MPA) = 27.47, and the combination index (CI) = 0.3362 < 1, which showed a synergistic effect on pharmacology (Figure 5d, 5e). Moreover, only when the reaction part of the system reaches an 0.8 < FA < 0.9, the synergistic effect will be reversed to the antagonistic effect, so the synergistic effect is stable (figure 5f).

A. Cell viability curve: the blue represents A2058R cells treated with vemurafenib, and the red represents 3-MPA. B. Cell viability curve: the blue represents A2058R cells treated with vemurafenib, and the red represents 3-MPA+vemurafenib. C. IC50 statistical plot: the yellow represents the A2058R cells treated with vemurafenib, the blue represents 3-MPA, and the greenrepresents 3-MPA+vemurafenib. IC50 in A2058R cells treated with 3-MPA+vemurafenib is lower than A2058R treated with 3-MPA or vemurafenib alone. D. Chou-Talalay model: the blue represents A2058R treated with 3-MPA, and the red and green represent those treated with vemurafenib and 3-MPA+vemurafenib respectively. E. Median-Effect Plot: Fa and Fu represent the reacted and the unreacted parts in the reaction system, respectively; The combination index (CI) = 0.3362 < 1 shows that the two drugs play a synergistic role. F. Combination index plot: The blue represents the therapy of 3-MPA+vemurefenib. When 0.8 < Fa < 0.9, the combination index (CI) > 1, and the combined effect turned into antagonism.

### Inhibition of PCK1 by 3-mercaptopicolinic acid led to ROS accumulation and oxidative damage in drug-resistant cells

**Figure 6.**
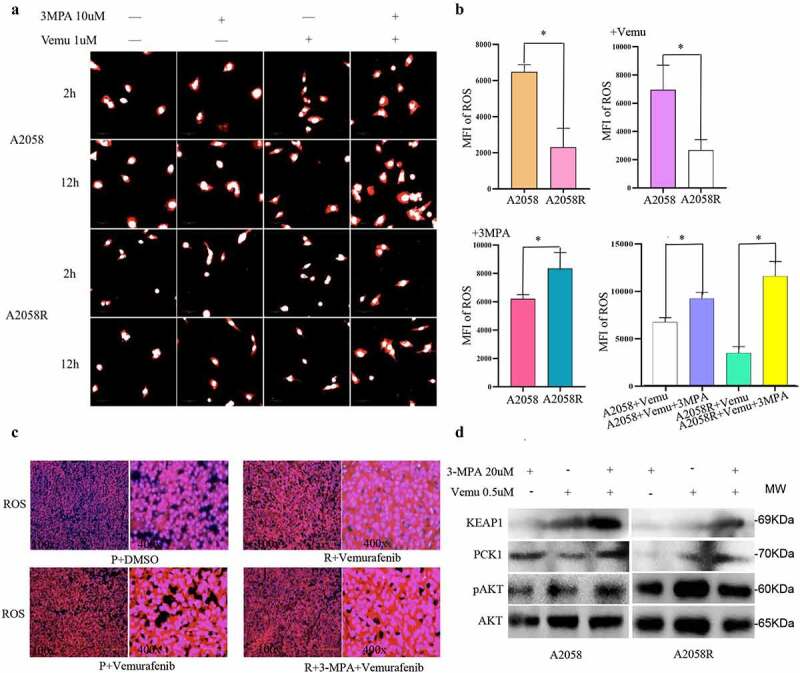
PCK1 led to drug resistance by reducing ROS accumulation.

PCK1 was a key enzyme in the PPP process which controls the levels of intracellular ROS. Therefore, we speculated that the downstream mechanism of PCK1 might be related to the regulation of ROS levels. We used the High Content Live Cell Imaging System to observe the level of ROS. As shown in Figure 6a, we found that the ROS in primary A2058 cells was significantly higher than that in A2058R (Figure 6b). When A2058R cells were exposed to vemurafenib, the level of ROS was still lower than that in A2058 cells. In addition, the levels of ROS in primary A2058 cells and drug-resistant A2058R cells increased after adding 3-MPA. When A2058 and A2058R cells were treated with 3-MPA+vemurafenib, the ROS increased significantly, which suggested that the level of ROS in drug-resistant cells was determined by the activity of PCK1.

A.ROS imaging: The red area is the result of ROS probe combined with intracytoplasmic ROS, and the white area represents the nuclear components. B. Result of ROS imaging. C. Detection of ROS in frozen tissue: the sliced tissues were derived from the subcutaneous tumorigenesis nude mice model. P represented the subcutaneous tumor formed after inoculation with melanoma A2058 cells, R represented the subcutaneous tumor formed after inoculation with drug-resistant A2058R cells, red represented the ROS, blue represented the nuclei (DAPI). The redder the color, the higher the content of ROS in the tissue. D. Western blot: KEAP1 expression increased after the addition of 3-mercaptopicolinic acid in A2058 and A2058R cells.

**Figure 7.**
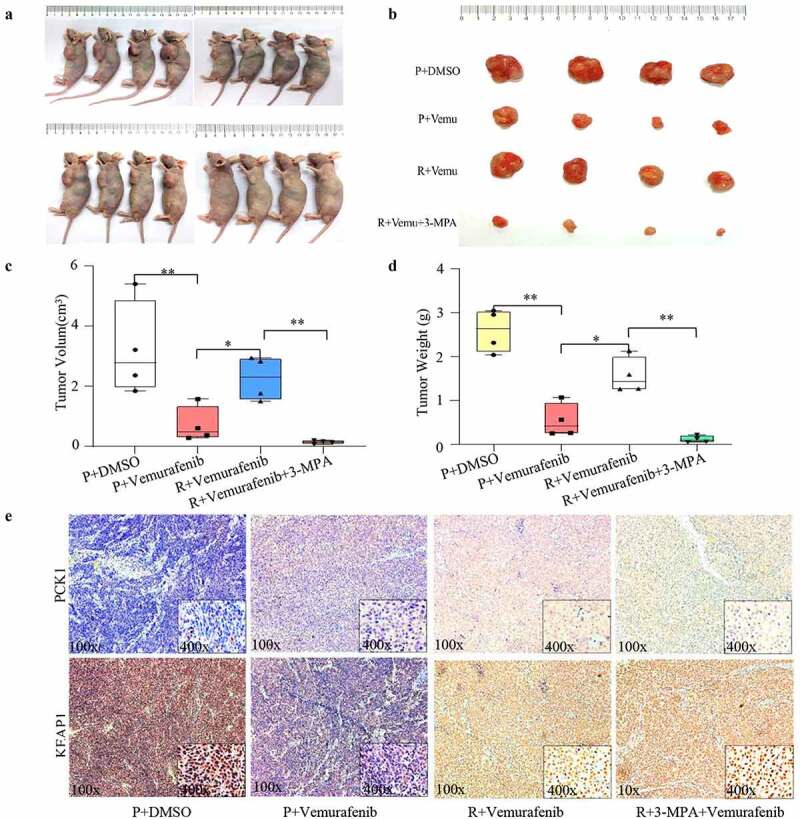
Verification of the chemoresistance mechanism of PCK1 and the benefit of combination therapy *in vivo.*

We generated a subcutaneous xenograft model from nude mice, forming cryosections, and analyzed by ROS probe (Figure 6c). Combined with the tumor volume (Figure 7d, 7e), we found that there were higher ROS in the A2058+ vemurafenib group than in the A2058+ DMSO group, whereas the number of tumor cells was reduced relative to the A2058+ DMSO group, indicating that vemurafenib inhibited progression by generating strong oxidative damage. Compared with the A2058R+vemurafenib group, the A2058+ vemurafenib group exhibited higher ROS but less cells, indicating that the drug-resistant cells hedge the killing effect of vemurafenib via regulation of ROS. Compared with the A2058R+vemurafenib group, the A2058R+3-MPA+vemurafenib group showed a significant increase of ROS but a decrease in the number of tumor cells, which demonstrated that the inhibition of PCK1 sensitized melanoma to vemurafenib. When we added 3-MPA (20 umol/L) to A2058 and A2058R cells, the WB results showed that the expression of KEAP1 was increased, which indicated that PCK1 inhibited the expression of KEAP1 (Figure 6d). 3-MPA blocked PCK1, thereby enabling ROS accumulation and suppressing chemoresistance in melanoma cells.

A. Nude mice model of subcutaneous tumorigenesis. B. Size of subcutaneous tumor tissues. C. Volume of tumor tissues: the combination group is the lowest. D. Weight of tumor tissues: the combination group is the lowest. E. Immunohistochemical results: the immunohistochemical staining of KEAP1 and PCK1 is shown in yellow; blue represents the nuclei (DAPI). In non-drug resistant tissues (P), the expression of PCK1 increased and that of KEAP1 decreased after using vemurafenib. In resistant tissues (R), the level of PCK1 was higher than that in P; the combination of 3-MPA+vemurafenib decreased the levels of PCK1 and increased the levels of KEAP1.

### *3-mercaptopicolinic acid improved the inhibitory effect of BRAFi* in vivo

Next, we evaluated the effect of combination therapy in melanoma using a subcutaneous xenograft mouse model (Figure 7a, 7b). According to the wt and volume data (Figure 7c, 7d), we found that the combination of vemurafenib and 3-mercaptopicolinic acid in the drug-resistant group could reverse the drug-resistant situation and significantly reduce the wet and volume of the tumor tissue. Compared with A2058 primary melanoma cells, the inhibition ability of the combination group was even more prominent, which suggested that the combination of BRAFi and PCK1 could achieve an excellent inhibition effect in resistant cells with PCK1 mutation. Combined with the immunohistochemical results, mentioned above, we further confirmed the main effect of 3-MPA was to increase ROS.

The immunohistochemistry results showed that in the subcutaneous tumor tissues of nude mice inoculated with primary A2058 cells, the expression of PCK1 in the vemurafenib group was up-regulated compared with the DMSO group, while KEAP1 was inhibited (Figure 6d). In the tissues of A2058R cells, the expression of PCK1 was limited, but the expression of KEAP1 was up-regulated. Therefore, both the *in vitro* and *in vivo* experiments confirmed that vemurafenib could induce high expression of PCK1, and then inhibit the synthesis of KEAP1. However, when 3-MPA was used to inhibit PCK1, KEAP1 was activated leading to ROS accumulated, which caused oxidative attack to resistant melanoma cells.

## Discussion

Acquired resistance is the main clinical obstacle to improving the prognosis of melanoma patients, and the known mechanisms involve various components [17,18]. The most common pathological mechanisms are the reactivation of the BRAF/MEK pathway or other proliferation promoting signal transduction pathways, the increasing copy number in the BRAF V600E protein , RAS mutations, and the activation of a-raf and c-raf, which promote the formation of a dimer that b-raf inhibitor cannot work[19,20]. Moreover, the mutation of MEK1/MEK2 and the high expression of COT lead to the activation of the MAPK/ERK pathway downstream of b-raf[21]. Overexpressed RTKs and the compensatory up-regulation of the Akt/PI3K/mTOR pathway contribute to the drug resistance [22,23]. Our study also demonstrated that the adaptive hyperactivation of AKT in response to BRAFi compensated for b-raf inactivation. Therefore, we concluded that the AKT/PI3K/mTOR pathway was an extremely important signaling axis, promoting cell proliferation, migration, and stemness, which contributed to BRAFi resistance in V600E mutated melanoma.

Although there are many downstream effectors of PI3K and Akt that can change the function of melanoma cells, PCK1 synthesis and activation are important processes mediated after AKT phosphorylation. The Akt/PI3K/mTOR pathway is mainly related to the mechanism of metabolic reprogramming[24], including the activation of mTOR complex 1 (mTORC1), glycogen synthase kinase 3 (GSK3), and forkhead box O (FoxO) transcription factor family members [25,26]. In our study, the activity of the Akt/PI3K/mTOR pathway and the PCK1 levels always paralleled each other *in vitro*, probably because the activation of mTORC1induced cytosolic SREBP to promote PCK1 synthesis[27]. Additionally, FoxO1 deacetylation synergized PGC-1α action to promote PCK1 transcription. It has recently been reported that activated Akt can directly phosphorylate PCK1 at Ser-90[28]. Ultimately, the biological processes mediated by PCK1 under the agonism of AKT are critical steps for drug resistance in melanoma.

A redox homeostasis imbalance elevates ROS levels and is pivotal in driving the processes of carcinogenesis, metastasis, and drug resistance in cutaneous melanoma [29,30]. Lim pointed out that was the process of redox metabolism rewiring in melanoma cells[31]. During the course of long-term treatment with BRAFi, the upregulated MITF-PGC1α axis and the long non-coding RNA (lncRNA) SAMMSON-p32 led to an inevitable accumulation of ROS[32]. High levels of ROS should have induced apoptosis, but genetic changes led to survive at high levels of ROS by increasing the NADPH level through the PPP or by activating the KEAP1 /Nrf2 antioxidant signaling pathway [33,34]. One of the crucial switches driving redox remodeling was PCK1, which was a key enzyme involved in the PPP process[35], playing a role in the antioxidant function of tumors[36]. This required the presence of PCK1 to synthesize abundant reductive NADPH via the PPP, and its inhibition impeded the growth of melanoma[37]. Moreover, PCK1 repressed KEAP1 and the Nrf2 restriction was removed, further activating the antioxidant repair ability of cells. Thus, a large amount of reductive NADPH was produced, which counteracted the cell damage induced by high levels of ROS, and promoted cells to achieving redox equilibrium to survive.

Studies on the role of redox fragility in melanoma progression have been reported, and BRAFi-resistant melanoma cells with elevated ROS levels are more sensitive to the oxidants[38]. This is highly consistent with our results, which shows that 3-mercaptopicolinic acid sensitizes the biological effect of vemurafenib and restores the sensitivity of tumors. When 3-mercaptopicolinic acid was used, drug-resistant cells lost the ability of supplementing the reduction equivalent, the redox level was unbalanced again, and the cells experienced oxidative damage. The specific mechanism underlying this effect might be that 3-mercaptopicolinic acid blocked the PPP to synthesize NADPH and nucleic acids, which resulted in inhibition of proliferation and l accumulation of intracellular ROS . Interestingly, the increase in ROS level should play a protective role through the KEAP1 /Nrf2 axis. However, when 3-mercaptopicolinic acid was used, the expression of KEAP1, a repressor of Nrf2, increased[39]. As the KEAP1 /Nrf2 axis had cellular protective effects on the antioxidant and detoxification activities[40], inhibiting Nrf2 undoubtedly aggravated the oxidative burden of tumor cells. These results suggested that 3-mercaptopicolinic acid not only down-regulates the PPP, but also indirectly up-regulates KEAP1, leading to the imbalance of antioxidant system in drug-resistant cells.As a result, the combination of 3-mercaptopicolinic acid and vemurafenib shows an outstanding therapeutic effect.

One limitation in our study is that only the melanoma A2058 cell line was used for modeling. Moreover, the drug resistance was a relatively long-term change, and the observation time used in our *in vitro* and *in vivo* experiments was relatively short. Therefore, the role of PCK1 in the drug resistance process of other tumor cells needed to be further revealed, and the observation cycle also needs to be optimized to explore the specific functions of PCK1 from a long-term and objective perspective.

## Conclusion

**Figure 8.**
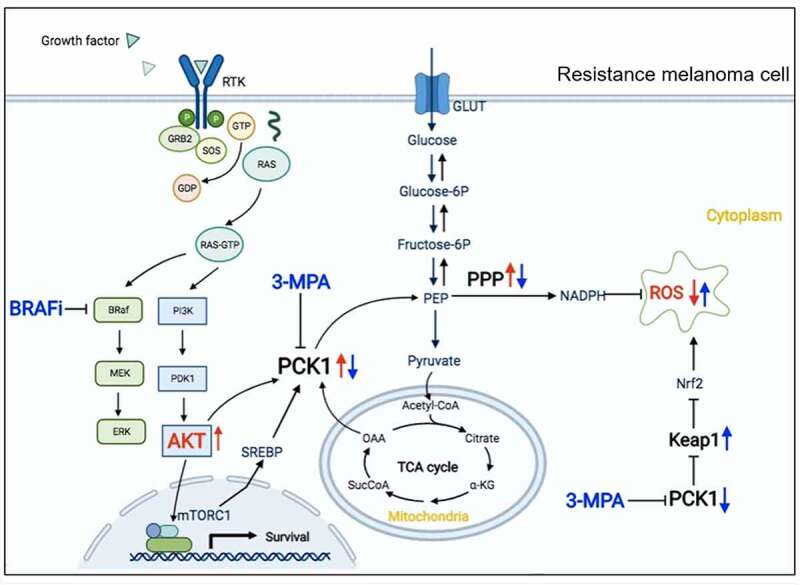
Mechanism of PCK1 and 3-mercaptopicolinic acid in resistant melanoma cells.

We found that the Akt/PI3K/mTOR signaling pathway was activated in resistant melanoma cells, and then PCK1 was upregulated in also promoted cell proliferation, migration, and tumor stemness. And then, PCK1 decreased the expression of KEAP1 and the leave of ROS (Figure 8). Based on the strategy of precision treatment, we used an antihyperglycemic agent 3-MPA combined with vemurafenib and found it enhanced the sensitivity of drugs obviously. Finally, we clarified the mechanism of resistance to vemurafenib and provided new options for clinically targeted combination therapy in patients with advanced melanoma.

In resistant melanoma cells to b-Raf inhibitors, the AKT/PI3K signaling pathway is activated, which promotes the synthesis of PCK1 and phosphorylated PCK1. On the one hand, it mediates the upregulation of the PPP metabolic pathway, synthesizes a large amount of NADPH, reduces the intracellular ROS. On the other hand, it inhibits KEAP1, releases the restriction of Nrf2, and activates the antioxidant effect of cells. All of these will promote the acquired drug resistance. Combining with 3-MPA, the synthesis of NADPH by the PPP will decrease, and the antioxidant activity of cells will be inhibited, and then lead to the accumulation of ROS. As a result, 3-MPA can strengthen the killing effect by making oxidative attack to resistant melanoma cells.

## Data Availability

The data used to support the findings of this study are available from the corresponding author on reasonable request.
